# Editorial: Acute liver failure in children

**DOI:** 10.3389/fped.2024.1402119

**Published:** 2024-04-03

**Authors:** Akash Deep, Pierre Tissieres

**Affiliations:** ^1^ Paediatric Intensive Care Unit, King’s College Hospital NHS Foundation Trust, London, United Kingdom; ^2^ Department of Women and Children’s Health, School of Life Course Sciences, King’s College London, London, United Kingdom; ^3^ Pediatric Intensive Care and Neonatal Medicine, Bicêtre Hospital, AP-HP Paris Saclay University, Le Kremlin-Bicêtre, Paris, France; ^4^ Institute of Integrative Biology of the Cell, CNRS, CEA, Paris Saclay University, Gif-sur-Yvette, France

**Keywords:** acute liver failure, intensive care, extracorporeal support, artificial liver-assist device, children

**Editorial on the Research Topic** Acute liver failure in children

Paediatric acute liver failure (PALF) is a rare condition associated with high morbidity and mortality. It is diagnosed in a child with no prior history of liver disease, who additionally has biochemical evidence of acute liver injury, and either an INR ≥2.0 with or without encephalopathy, or an INR ≥1.5 in the presence of hepatic encephalopathy despite treatment with Vitamin K (1). In addition to being a rare disease, aetiology of ALF in children is varied and they differ in different age-groups as well making accurate diagnosis and management challenging (1). Despite advanced investigations, we do not have a confirmed diagnosis in 35%–45% children with ALF (indeterminate aetiology). In recent years, non-transplant measures with high quality intensive care has increased the survival of children without the need for transplantation (transplant-free survival) (2). Early transfer to liver transplant centre, starting supportive measures especially aetiology specific treatment, maintaining euglycemia and sodium levels, use of antimicrobials, use of extracorporeal therapies [Continuous renal replacement therapy (CRRT), total plasma exchange, albumin dialysis], strict neuromonitoring and neuroprotection form the main stay of treatment in children with ALF (2–7) Liver transplantation forms the mainstay of treatment if medical management fails or for selected aetiologies especially indeterminate causes where aetiology specific treatment cannot be started.

The goals of this collection of 5 manuscripts are to provide the readers with a varied presentation and treatment options in children with acute liver failure in both the low-middle income countries (LMIC) and high income countries. This collection shows how challenging the diagnostic and management strategies can in options can be in patients with ALF.

In the “*Case Report: Fatal Acute Liver Failure With Giant Cell Transformation in a Pediatric Patient Associated With MIS-C*” the authors from Columbia describe a diagnostic conundrum when they treated a 10-month child with MIS-C associated acute liver failure. After ruling out infectious, metabolic, genetic, and autoimmune causes of liver failure, authors got the positive results for IgM and IgG for SARS-CoV-2 and negative RT-PCR test for SARS-CoV-2. This fitted in the diagnosis of COVID-19-related paediatric multisystem inflammatory syndrome (MIS-C) (8). Despite all supportive measures for ALF and immunomodulation, the child unfortunately died. This case emphasised the importance of thorough investigations in children with ALF and the diverse presentation of MIS-C.

In the “*Association of duration and aetiology with the effect of the artificial liver support system in paediatric acute liver failure*” authors from China assessed the efficacy of the artificial liver support system (ALSS) in 39 children with acute liver failure and risk factors associated with the effect of ALSS. The most commonly used modality of ALSS was plasma exchange combined with continuous renal replacement therapy. The overall survival rate was 76.9%. 38.4% patients received only one modality, whereas 61.6% patients received hybrid treatments. Authors concluded that ALSS could effectively reduce serum bilirubin, alanine aminotransferase and serum ammonia levels which depended on the underlying aetiology. Longer the duration of ALSS, more effective was ALSS in reducing ammonia concentration.

In the “*Pediatric acute liver failure: An experience of a pediatric intensive care unit from resource limited settings*” authors from South India retrospectively looked at the etiologies, outcome and prognostic factors in 125 children with ALF and the validity of the existing liver transplantation criteria to predict the outcome of PALF in resource limited settings.. Very interestingly, of 125 children with acute liver failure, the main etiologies were infections (32%), indeterminate (23%), paracetamol toxicity (21%), metabolic (13%) and others (11%). Dengue was the most common infection (55%). This showed the difference in aetiologies between LMIC and HIC where metabolic, drug induced aetiologies predominate. Of 125 patients, 63.2% (*n* = 79) had spontaneous regeneration which was higher in paracetamol induced (92.3%) compared to non-paracetamol induced acute liver failure (55.5%). Only two patients underwent liver transplantation and 35% died. Authors found that INR >4 was more sensitive than King's College Criteria for predicting the need for liver transplantation. Authors felt that perhaps there was a need for a better prediction model for outcomes of PALF in resource limited settings.

In the “*Case report: Acute liver failure in children and the human herpes virus 6-? A factor in the recent epidemic*” authors from Birmingham, UK shed light on the hepatitis outbreak which took place in 2022 in the western world. A number of series have been published implicating adenovirus subtype-41-F, SARS-CoV2, in severely affected children, especially in those requiring liver transplantation (LT). In UK case series of the recent hepatitis epidemic, blood and/or liver samples sent for metagenomic analysis all tested positive for human herpes virus 6 (HHV-6) with another series reporting positivity for adenovirus (9). Authors describe the clinical course of three female infants with acute hepatitis and ALF who progressed to LT, in whom primary HHV-6 infection was suspected. This series emphasised the importance for routine screening for HHV-6 in children with acute hepatitis and the use of effective HHV-6 anti-viral prophylaxis to prevent recurrence post-transplant.

In the last manuscript in this series “*Clinical application of regional citrate anticoagulation for membrane-based therapeutic plasma exchange (mTPE) in children with liver failure*” authors from China have addressed a very important but controversial aspect of safety and efficacy of anticoagulation with regional citrate (RCA) where there is a danger of citrate accumulation in PALF. Authors had used the total to ionized calcium ratio (T/iCa) >2.5 as the diagnostic criteria for citrate accumulation (CA). Similar to previous reports showing safety of RCA in CRRT for ALF, authors of this manuscript concluded that though children undergoing membrane-TPE are at risk for developing hypocalcaemia and citrate accumulation, with proper protocol adjustment, however, RCA-mTPE can be used safely and effectively in these patients (10).

Therefore, in the rare but life-threatening condition, all efforts should be made to find an aetiology causing ALF as aetiology specific treatment forms the cornerstone of treatment in PALF. Extracorporeal therapies—CRRT and TPE need to be started on time and circuits should be anticoagulated to keep the downtimes low. Regional citrate anticoagulation, though thought to cause citrate accumulation can be safely and effectively used in this cohort. With all the supportive measures in place and high-quality intensive care, transplant-free survival is getting better.
